# Needs of forensic psychiatric patients with schizophrenia in five European countries

**DOI:** 10.1007/s00127-022-02336-5

**Published:** 2022-07-15

**Authors:** R. Oberndorfer, R. W. Alexandrowicz, A. Unger, M. Koch, I. Markiewicz, P. Gosek, J. Heitzman, L. Iozzino, C. Ferrari, H.-J. Salize, M. Picchioni, H. Fangerau, T. Stompe, J. Wancata, G. de Girolamo

**Affiliations:** 1 Clinical Division of Social Psychiatry, Medical University of Vienna, Währinger Gürtel 18-20, 1090 Vienna, Austria; 2 Institute of Psychology, University of Klagenfurt, Klagenfurt, Austria; 3 Institute of Psychiatry and Neurology, Department of Forensic Psychiatry, Warsaw, Poland; 4 Unit of Epidemiological Psychiatry and Evaluation, IRCCS Istituto Centro San Giovanni Di Dio Fatebenefratelli, Brescia, Italy; 5 Medical Faculty Mannheim, Central Institute of Mental Health Mannheim, Heidelberg University, Heidelberg, Germany; 6 Department of Forensic and Neurodevelopmental Science, Institute of Psychiatry, Psychology and Neuroscience, King’s College, London, UK; 7 St Magnus Hospital, Haslemere, Surrey UK; 8 Department of the History, Philosophy and Ethics of Medicine, Medical Faculty, Heinrich-Heine-University, Duesseldorf, Germany

**Keywords:** Forensic psychiatry, Schizophrenia spectrum disorders, needs assessment, Health service research

## Abstract

**Aims:**

The purpose was to compare the frequency of needs of patients with schizophrenia in forensic services across five European countries as assessed by both the patients and their care staff.

**Methods:**

Patients with schizophrenia and a history of significant interpersonal violence were recruited from forensic psychiatric services in Austria, Germany, Italy, Poland and England. Participants’ needs were assessed using the Camberwell Assessment of Needs—Forensic Version (CANFOR). Multiple linear regression analyses were used to identify predictors of numbers of needs.

**Results:**

In this sample, (*n* = 221) the most commonly reported need according to patients (71.0%) and staff (82.8%) was the management of psychotic symptoms. A need for information was mentioned by about 45% of staff and patients. Staff members reported a significantly higher number of total needs than patients (mean 6.9 vs. 6.2). In contrast, staff members reported a significantly lower number of unmet needs than patients (mean 2.0 vs. 2.5). Numbers of total needs and met needs differed between countries. Unmet needs as reported by patients showed positive associations with the absence of comorbid personality disorder, with higher positive symptom scores and lifetime suicide or self-harm history. Significant predictors of unmet needs according to staff were absence of comorbid personality disorder and higher positive as well as negative symptom scores according to PANSS.

**Conclusions:**

Staff rated a significantly higher number of total needs than patients, while patients rated more unmet needs. This indicates that patients’ self-assessments of needs yield important information for providing sufficient help and support.

**Supplementary Information:**

The online version contains supplementary material available at 10.1007/s00127-022-02336-5.

## Introduction

One percent of the population will develop a schizophrenia or a similar psychotic disorder during their lifetime. Several studies show an increased risk of committing violent crimes among patients with schizophrenia compared to persons without this disorder. A systematic review [1] demonstrated a clear association between schizophrenia, comorbid substance use disorders and violence. As a consequence, patients with schizophrenia are over-represented in forensic services compared to patients with other mental disorders.

Forensic psychiatric services are generally considered as the most appropriate treatment setting for people who have committed an offence while suffering from a mental disorder. Nevertheless, there exist relevant differences between different European countries regarding forensic services. Several authors [2] showed differing prevalence rates of forensic psychiatric patients and of forensic beds for some European countries: Salize and colleagues [3] reported for Germany 7.5, for England and Wales 5.7, for Austria 3.4 and for Italy 2.2 forensic psychiatric patients per 100,000 of the population, but no rates for Poland. Tomlin and colleagues [4] found in their recent international study similar rates for Germany (10.0/ 100000), higher rates for England and Wales (11.7/100000), and slightly lower rates for Italy (1.7/100000). For Poland they reported 5.7 patients per 100,000 of the population, but no data for Austria. Countries differ with respect to the average length of stay in forensic services with 7.9 years in Germany, 7.4 years in England and Wales, 2.9 years in Italy and 2.0 years in Poland (no data for Austria [4]). In England and Wales the proportion of female inpatients in forensic care was much higher (18%) than in Italy (8%) and Poland (7% [4]). Unfortunately, all studies reporting data on forensic services cover only selected countries, but not all European countries [3–5]. This limits all comparisons between European countries. Although some authors assume differences in data reporting practices across jurisdictions [6], we must assume that there are true differences between forensic services in different European countries.

About 30 years ago, several authors suggested to plan services based on patients’ needs [7–10]. Of course, each patient needs a different combination of interventions. Thus, for service planning it is difficult to determine which interventions are necessary and how often. To assess patients’ needs, several instruments were developed (e.g., Needs for Care Assessment [7], Camberwell Assessment of Need [11], Cardinal Needs Schedule [12] allowing to plan psychiatric services based on a “bottom-up” approach. Brewin et al. [13] suggest that, in principle, each assessment of needs should be based on a close evaluation of current psychiatric symptoms and the patient’s social functioning. This implies that needs are defined and assessed by psychiatric experts. Other authors emphasized that the assessment of needs should be complemented by the subjective views of patients, and pointed out that in everyday clinical practice patients are involved in negotiations as to what interventions should be provided for which problems. These authors emphasized that patients’ subjective views are as important as the staff’s views and, therefore, should be rated separately from the latter [14].

Harty et al. [15] reported that forensic psychiatric patients have a greater number of unmet needs compared to the general psychiatric population. In an Italian sample [16], needs most frequently reported among forensic patients were information about the illness, getting enough food, daytime activities, psychotic symptoms and intimate relations, while in England [15] receiving benefits, getting enough food, looking after the home and self-care were most common. In the Italian study [16] getting enough food was met by 100% and help for psychotic symptoms by 78%, while need for intimate relations was unmet by more than 80%. Need for daytime activities was unmet among 58% and information about the illness was unmet among 46%. In the English sample, [15] the need for receiving benefits, getting enough food, looking after the home and self-care was met by more than 98%. Such differences between studies indicate the wide range of help provided to forensic psychiatric patients. Some authors [14, 15] pointed out that forensic patients have specific needs which should be specifically assessed. Risks for arson and for sexual offending are common problems among patients of forensic psychiatric services. Forensic patients suffering from schizophrenia often have an increased risk for violence which must be considered using pharmacological as well as non-pharmacological strategies.

Assessing these needs in a standardized way is essential, both for the delivery of effective treatment and for the development of tailored services [16]. The tailored provision of treatment for forensic psychiatric patients would have far-reaching consequences. If needs of forensic patients are considered aptly, resources could be more efficiently targeted. This could contribute to more effective psychiatric and social interventions allowing for earlier discharge from forensic inpatient services and reduction in the risk of reoffending. Unmet needs together with severe mental disorders are major problems from the perspective of detainees, some of the needs have been identified as risk factors for criminal offending [17].

The literature on needs assessment of forensic patients including their own perceived needs is scarce. Some studies have analysed frequencies of needs regarding sociodemographic and clinical aspects such as age, gender, length of illness or different types of forensic services [15, 18, 19]. While some authors reported differences, others did not find significant differences between groups. Considering that the availability of forensic psychiatric services differs between countries, we must assume that needs will differ between countries. Unfortunately, we did not find studies comparing needs in different countries using diagnostically homogeneous samples.

### Aims

The purpose of this study was to investigate the type and frequency of needs in forensic services of patients with schizophrenia spectrum disorders across five European countries as assessed by the patients themselves and their care staff. Further, we aimed to analyse if the overall frequency of met, unmet and total needs differs between countries. Finally, we planned to analyse, whether predictors of the overall frequencies of met, unmet and total needs can be identified.

## Methods

### Sample

The present study is part of the “European Study on Risk Factors for Violence in Mental Disorder and Forensic Care (EU-VIORMED)” [20]. Patients with a primary diagnosis of a Schizophrenia Spectrum Disorder (SSD) according to DSM-5 [21] and a history of significant interpersonal violence were recruited from several forensic psychiatric institutions in Austria, Germany, Italy, Poland and England (see table S1). Significant interpersonal violence was defined as having committed homicide, attempted homicide or other assault which had caused serious physical injury to another person.

Diagnoses were made by the treating clinicians. All subjects were between 18 and 65 years of age. The main exclusion criteria were: (i) confirmed intellectual disability; (ii) traumatic brain injuries or organic brain disorders; (iii) not being able to speak the national language fluently. A questionnaire including the specific recruitment criteria was provided to identify patients.

In each study centre, treating clinicians invited patients under their care to participate in the study. Participants were provided with written information about the study and had an opportunity to ask questions. If they agreed to participate, they were asked to provide written informed consent before starting the assessment. Informed consent was also sought to allow to collect information from caregivers, family members or case-managers/ clinical staff for additional/missing information.

Initial plans were to recruit at least 200 patients overall with similar proportions in each country. However, the worldwide coronavirus outbreak and the resulting restrictions after February 2020 limited the recruitment in some countries. The degree and impact of the restrictions varied between the five countries and affected recruitment.

The study was approved by the Ethics Committees of each participating site. All participants provided written informed consent before entering the study after a full verbal and written description of the study’s aims and methods.

### Assessments

All subjects were evaluated by research workers employed by the study and centrally trained on each instrument. Socio-demographic, core clinical, and criminological data were collected based on patient interviews and later cross-referenced with medical and criminal records and clinician review. DSM-5 diagnoses were based on treating clinicians’ evaluations extracted from the medical records.

Participants’ needs were assessed by means of the research version of the Camberwell Assessment of Needs—Forensic Version (CANFOR [22]), which is the most commonly used research instrument for this purpose targeted to forensic patients [23]. The CANFOR assesses patients’ needs across 25 domains covering basic living skills, mental and physical health, social, functional and service need areas. It is based on the non-forensic version of the Camberwell Assessment of Need (CAN [11]), which covers 22 domains. The CANFOR adds three additional domains specifically targeted at forensic patients: treatment, sexual offences and arson [22]. Furthermore, it includes patient ratings as well as staff ratings [22]. CANFOR defines a need as being present when there have been difficulties in a particular area over the last month [24]. The interviewer first evaluates whether a need is present or not and if present, whether that need is met or unmet. A met need is defined as a difficulty for which an appropriate intervention is currently being received from either formal or informal sources. An unmet need is defined as a difficulty for which no interventions are currently being provided by local services, or that interventions provided are not perceived as effective. The numbers of met and unmet needs can be summarized into a met needs score and an unmet needs score. The total need score is the sum of the number of met needs and unmet needs [22]. The CANFOR has established reliability and validity [24].

Current psychopathological symptom severity was assessed using the Positive and Negative Syndrome Scale-PANSS [25], based on a semi-structured patient interview and clinical observation. PANSS scoring used the original standard PANSS model [25]. PANSS items were summarized into three sub-scales (positive, negative and general).

### Statistical analyses

The composition of the sample was described using descriptive statistics. The frequencies of single CANFOR items were reported as percentages. For CANFOR sum-scores means and standard deviations are given. To analyse, whether CANFOR sum-scores differ between the five European countries, univariate analyses of variance were performed.

To identify predictors of CANFOR sum-scores several multiple linear regression analyses were performed. We planned to include the following independent variables: age, sex, comorbidity with personality disorder, type of SSD (schizophrenia vs. others), highest occupational status, lifetime ever attempted suicide or self-harm, lifetime substance or alcohol use, PANSS scores (positive, negative, general), duration of illness, type of index offence (attempted homicide vs. others), presence of any other violent behaviour lifetime (in addition to index offence), age at first contact with mental health services, time since first admission to a forensic unit, number of lifetime admissions to forensic units, and five European countries. Since the numbers of needs have a skew distribution and the sample variance is clustered at the country level, robust standard errors were estimated for correct assessment of statistical inference. To detect collinearity, correlations between all independent variables were performed: for this reason, we had to exclude age because it was highly correlated with several other variables, and the PANSS general score because it was highly correlated with the PANSS negative and PANSS positive score from regression analyses.

For all analyses, a critical alpha of 0.05 was considered as significant.

## Results

### Socio-demographic, clinical and forensic characteristics

Of 321 forensic patients with a primary diagnosis of SSD who were invited to participate in this study, 221 (69.2%) participated and 99 (30.8%) refused. The country distribution is shown in Table 1. The mean age of the sample was 39.22 years and the large majority was of male sex. More than three quarters suffered from schizophrenia and more than a quarter had a diagnosis of a comorbid personality disorder. Nearly half of the sample was detained because of homicide or attempted homicide. The mean duration of illness was about 13 years and the mean time since the first admission to a forensic unit was more than 6 years. While the mean age was similar in all five country samples (see table S2), the time since first admission to a forensic unit was 9.65 years in England and only 3.97 years in Italy. In Italy nearly 80% had (attempted) homicide as index offence and in England this proportion was less than a quarter. The PANSS scores showed a broad range between countries with highest mean values in Poland and lowest mean values in Germany. While in Poland only 17.9% were diagnosed with a comorbid personality disorder, this rate was more than 30% in Austria, Italy and England.

**Table 1:** Sample description (*n* = 221; SD = standard deviation)

Country	
Austria	50 (22.6%)
Germany	36 (16.3%)
Italy	39 (17.6%)
Poland	56 (25.3%)
England	40 (18.1%)
Sex	
Male	195 (88.2%)
Female	26 (11.8%)
Age (years)	
Mean (SD)	39.22 (11.18)
Highest occupational status	
Unskilled	146 (66.1%)
Skilled/professional	75 (33.9%)
Type of Schizophrenia Spectrum Disorder	
Schizophrenia	174 (78.7%)
Other	47 (21.3%)
PANSS positive score	
Mean (SD)	14.80 (6.89)
PANSS negative score	
Mean (SD)	18.90 (7.73)
PANSS general score	
Mean (SD)	33.86 (11.22)
Comorbidity with personality disorder	
No	152 (70.7%)
Yes	63 (29.3%)
Lifetime substance or alcohol use	
No	50 (22.7%)
Yes	170 (77.3%
Lifetime ever attempted suicide(s) or self-harm	
No	119 (54.1%
Yes	101 (45.9%)
Duration of illness (years)	
Mean (SD)	13.23 (9.60)
Type of crime (index offence)	
(Attempted) homicide	104 (47.1%)
Other	117 (52.9%)
History of any other violent behaviour lifetime (in addition to index offence)	
No	70 (32.3%)
Yes	147 (67.7%)
Age at first contact with mental health services (years)	
Mean (SD)	25.02 (9.11)
Time since first admission to a forensic unit (years)	
Mean (SD)	6.24 (6.92)
Number of lifetime admissions to forensic units	
Mean (SD)	1.50 (1.26)

### Frequency of met, unmet and total needs

The most common needs according to staff and patients were related to psychotic symptoms and daytime activities which were reported more often by the staff (Table 2). Need for getting information was mentioned by similar proportions of staff and patients. More than 40% of patients reported needs regarding maintaining and/or establishing intimate relationships (44.3%) and sexual expression (46.6%), whereas these problems were mentioned less frequently by staff (26.2% and 17.6%, respectively). For most domains, needs were more often met than unmet as assessed by both patients and staff.

**Table 2:** Frequency of needs according to CANFOR as assessed by patients and staff members

CANFOR items	Patient			Staff		
	Met need	Unmet need	Total need	Met need	Unmet need	Total need
	(%)	(%)	(%)	(%)	(%)	(%)
1 Accommodation	12.2	12.2	24.4	15.8	7.7	23.5
2 Food	26.2	8.6	34.8	30.3	5.0	35.3
3 Living environment	12.7	3.6	16.3	23.5	11.3	34.8
4 Self-care	4.1	3.2	7.2	13.6	8.6	22.2
5 Daytime activities	27.1	19.0	46.2	45.2	12.7	57.9
6 Physical health	28.1	10.0	38.0	33.0	7.7	40.7
7 Psychotic symptoms	59.3	11.8	71.0	57.9	24.9	82.8
8 Information	29.0	17.2	46.2	39.4	5.9	45.2
9 Psychological distress	27.1	12.7	39.8	29.4	14.5	43.9
10 Safety to self	2.3	4.5	6.8	5.9	3.2	9.0
11 Safety to others	8.1	4.1	12.2	16.3	10.0	26.2
12 Alcohol	7.7	1.4	9.0	17.2	3.6	20.8
13 Drugs	12.7	6.8	19.5	13.6	14.0	27.6
14 Company	15.8	11.3	27.1	22.2	12.2	34.4
15 Intimate relationships	7.7	36.7	44.3	9.0	17.2	26.2
16 Sexual expression	5.9	40.7	46.6	6.3	11.3	17.6
17 Child care	2.3	7.7	10.0	1.4	8.1	9.5
18 Basic education	7.7	3.6	11.3	8.1	2.3	10.4
19 Telephone	19.0	1.8	20.8	21.7	0.0	21.7
20 Transport	2.3	1.8	4.1	4.5	1.4	5.9
21 Money	18.6	10.9	29.4	28.1	7.7	35.7
22 Benefits	11.8	14.9	26.7	17.2	5.0	22.2
23 Treatment	25.8	6.3	32.1	31.2	3.6	34.8
24 Sexual offences	0.5	0.0	0.5	0.9	1.8	2.7
25 Arson	0.0	0.0	0.0	0.5	0.5	0.9

Staff members reported a significantly higher number of needs than patients themselves (mean 6.92 vs. 6.24, Table 3). While staff members assessed significantly more needs as met, patients classified more needs as unmet. In the England, the mean total numbers of needs were 10.5 according to patients and 8.03 according to staff, but in Poland only 4.12 and 5.89, respectively (Table 4). Similarly, large differences between countries were found regarding unmet and met needs. When performing analyses of variance, the mean numbers of unmet, met and total needs differed between countries according to both patients’ and staff assessment (Table 4). The mean numbers of needs according to the variables used for sample description are given in table S3.

**Table 3:** Mean of CANFOR sum-scores and t-test for comparing patient and staff ratings (SD = standard deviation)

CANFOR	Patient		Staff		*t*	*df*	*p*
	Mean	SD	Mean	SD			
Number of met needs	3.74	2.10	4.92	2.91	− 6.088	220	0.000
Number of unmet needs	2.51	2.69	2.00	2.13	2.996	220	0.003
Number of total needs	6.24	3.69	6.92	3.68	− 2.483	220	0.014

**Table 4:** Mean of CANFOR sum-scores in 5 European countries and analyses of variance for differences between countries (SD = standard deviation)

Country	Patient						Staff					
	Met needs		Unmet needs		Total needs		Met needs		Unmet needs		Total needs	
	Mean	SD	Mean	SD	Mean	SD	Mean	SD	Mean	SD	Mean	SD
Austria	3.48	1.83	2.32	2.13	5.8	2.81	4.22	3.19	2.36	2.20	6.58	3.89
Germany	3.17	1.78	1.36	1.20	4.53	1.76	4.36	3.53	1.50	1.92	5.86	4.59
Italy	4.54	2.08	2.54	2.05	7.08	3.29	7.38	2.42	1.31	1.81	8.69	3.12
Poland	3.02	2.07	1.11	1.36	4.12	2.95	4.16	2.06	1.73	1.73	5.89	2.75
England	4.8	2.14	5.7	3.55	10.5	3.58	4.97	2.08	3.05	2.58	8.03	3.36
Analysis of variance for differences between countries												
F	7.24		29.80		32.07		10.56		4.85		5.52	
p	0.000		0.000		0.000		0.000		0.001		0.000	

### Predictors of needs

Multiple linear regression analyses were used to identify predictors of met, unmet and total needs. Due to collinearity, age and the PANSS general score had to be excluded from regression analyses.

Patient reports of met needs were significantly positively associated with country (Italy vs. Austria, England vs Austria) and the history of any other violent behaviour during lifetime in addition to the index offence (Table 5). Unmet needs as reported by patients showed positive associations with England vs Austria, PANSS positive score and lifetime suicide attempts or self-harm. Unmet needs according to patients were negatively associated with Poland vs. Austria, female gender and comorbid personality disorder. Total needs were more common in Italy and England and less common in Poland as compared to Austria. Presence of comorbid personality disorder was negatively associated with total needs.

**Table 5:** Multiple linear regression with CANFOR sum-scores of patient and of staff assessment as dependent variables (beta = standardized regression coefficient)

	Patients						Staff					
	Number of met needs		Number of unmet needs		Total number of needs		Number of met needs		Number of unmet needs		Total number of needs	
Predictors	beta	*p*	beta	*p*	beta	*p*	beta	*p*	beta	*p*	beta	*p*
(Intercept)	− 0.18	**0.001**	− 0.09	0.201	− 0.16	< 0.001	− 0.38	0.062	0.1	0.513	− 0.25	0.050
Female vs. male	0.01	0.950	− 0.16	0.007	− 0.11	0.118	0.26	< 0.001	− 0.01	0.849	0.20	0.001
Highest occupational status skilled vs. unskilled	0.00	0.982	− 0.03	0.641	− 0.02	0.731	0.01	0.868	− 0.08	0.254	− 0.04	0.548
Other psychotic vs. schizophrenia	0.05	0.521	− 0.06	0.404	− 0.02	0.770	0.03	0.676	0.01	0.881	0.01	0.823
PANSS positive score	− 0.09	0.217	0.21	0.030	0.10	0.189	0.04	0.568	0.28	0.001	0.20	0.002
PANSS negative score	0.04	0.662	− 0.04	0.587	− 0.01	0.922	0.20	0.009	0.21	0.008	0.29	< 0.001
Presence of comorbid personality disorder	− 0.05	0.580	− 0.15	**0.013**	− 0.14	0.038	− 0.06	0.399	− 0.14	**0.035**	− 0.13	0.042
Lifetime substance or alcohol use	− 0.10	0.240	0.02	0.763	− 0.04	0.526	0.04	0.527	0.04	0.548	0.06	0.361
Lifetime ever attempted suicide(s) or self-harm	− 0.01	0.893	0.15	0.013	0.10	0.096	0.04	0.513	0.12	0.079	0.11	0.081
Duration of illness	0.08	0.381	0.01	0.949	0.05	0.542	− 0.01	0.894	− 0.01	0.920	− 0.01	0.859
Index violence (attempted) homicide	0.06	0.485	-0.04	0.549	0.00	0.942	− 0.01	0.834	− 0.10	0.133	− 0.08	0.258
History of any other violent behaviour lifetime (in addition to index offence)	0.20	0.012	0.01	0.825	0.12	0.059	0.19	**0.003**	0.09	0.133	0.21	0.001
Age at 1st contact with mental health service	− 0.04	0.596	0.10	0.102	0.05	0.400	− 0.06	0.405	0.10	0.173	0.01	0.871
Time since first admission to a forensic unit	− 0.09	0.372	− 0.07	0.465	− 0.10	0.226	− 0.06	0.486	0.03	0.806	− 0.03	0.743
Number of lifetime admissions in forensic units	0.00	0.969	0.13	0.276	0.09	0.279	0.06	0.506	− 0.16	0.210	− 0.05	0.423
Country Germany vs. Austria	− 0.11	0.614	− 0.26	0.123	− 0.25	0.104	0.35	0.146	− 0.21	0.331	0.15	0.500
Country Italy vs. Austria	0.62	0.011	0.16	0.382	0.48	0.020	1.55	< 0.001	− 0.26	0.198	1.08	< 0.001
Country Poland vs. Austria	− 0.19	0.346	− 0.43	0.003	− 0.43	0.011	− 0.04	0.819	− 0.37	0.052	− 0.26	0.125
Country England vs. Austria	0.66	0.006	1.10	< 0.001	1.18	< 0.001	0.23	0.266	0.43	0.071	0.44	0.029
R^2^	0.159		0.432		0.412		0.345		0.331		0.401	

Bold numbers indicate significant values

Staff reports of met needs were more common in Italy vs. Austria, women, among those with higher PANSS negative scores and those with any violent behaviour during lifetime in addition to the index offence. Unmet needs according to staff were positively associated with PANSS positive and PANSS negative scores, but negatively with comorbid personality disorder. Significant predictors of total needs according to staff were Italy and England vs Austria, female gender, PANSS positive and PANSS negative scores, violent behaviour in addition to the index offence, and the absence of comorbid personality disorder.

## Discussion

To our knowledge, this is the first study investigating the needs of patients with SSD, who have committed a violent offence and were treated in forensic units in multiple European countries.

In the overall sample, patients and staff reported a mean total number of needs (regardless if met or unmet) between 6 and 7. Other studies using the CANFOR reported mean scores between 3.4 [26] and 23.1 [27], most studies between 6 and 12 [15, 28–32]. Thus, all country subsamples are in the range of these existing studies. While some studies found higher numbers of met needs than of unmet needs [18, 28, 33], others reported more unmet needs than met needs [31, 32].

When comparing how often single CANFOR domains were unmet, some items were rated by patients markedly more often than by staff (e.g. intimate relationships, sexual expression, information, benefits, daytime activities). It may not be surprising that domains such as intimate relationships or sexual expression are better recognized by patients themselves; moreover, they are notoriously difficult to be met in closed, restrictive settings such as forensic units. Needs with a clinical content such as psychotic symptoms, drug or alcohol problems were reported more often by staff. Considering sum-scores, staff rated a significantly higher number of total and of met needs than patients themselves. In contrast, patients rated more unmet needs. Nearly all studies reported that staff assessed more total needs than patients [31, 34, 35]. While in some studies patients reported higher numbers of unmet needs than staff [30, 32], others reported higher numbers according to staff [35]. Segal et al. [30] hypothesized that higher numbers of unmet needs among patients might exist regardless of current interventions being provided in the inpatient setting. They conclude that this highlights the importance of ascertaining patient as well as staff perspectives, and sharing of different perspectives may enhance the patient’s engagement in treatment and thus contribute to better outcomes [3].

### Country differences in met and unmet needs

We found that the numbers of met, unmet and total needs differed significantly between the five European countries participating in our study. Some authors reported differences due to type of forensic service [36] and others due to the security level of the service [26, 33]. Since definitions of services and of security level vary with respect to national laws and regional structures, it is difficult to classify the services of our study. In addition, the capacity of forensic services, in terms of number of beds per capita, is likely to affect the casemix in forensic services and thus the amount of met, unmet and total needs. Unfortunately, the comparability of such data between countries show considerable methodological limitations [3].

### Can we predict met and unmet needs of forensic patients?

Multiple linear regression analyses were used to identify predictors for the number of needs. The fact that the significant predictors of the number of needs differed considerably between patients’ and staff rating indicates different perspectives of the two groups. This assumption applies in a similar fashion when analysing the various CANFOR domains separately.

Staff identified significantly more met and total needs for women than for men. This is in agreement with the studies of Harty and colleagues [15] as well as Long and colleagues [29]. More severe positive and negative symptoms predicted higher needs according to staff, and this suggests that professionals assign a stronger weight to clinical aspects of needs than patients. Similar to our study, Völlm et al. [37] found more unmet needs among those with a history of self-harm or suicide attempts. Persons with comorbid personality showed less total needs and unmet needs, which could indicate that this subgroup had received more support in the past. Numbers of total needs and of met needs differed significantly between countries according to staff and patients themselves. The Italian and the English sample reported higher numbers of needs that the Austrian sample. Since Italy has more, and Italy less forensic beds than Austria this difference cannot explain the increased needs in these countries [3]. Since gender was included into our regression analyses, differing gender distribution cannot have caused discrepancies between countries. Unfortunately, we do not have sufficient data regarding the length of stay in forensic services to interpret these findings.

### Limitations and strengths

Similar to other studies, we included forensic services which were willing to participate in this study. Since we do not know which factors influenced the preparedness of the heads of these services, we must not exclude that the selection of services was biassed. Another limitation of our study is the fact that we had no possibility to compare in detail security level of forensic services in the five countries. Further, we could not take into account the different legal frameworks and numbers of forensic beds in our analyses. Nevertheless, the inclusion of samples of five European is a strength of this study.

The restrictions associated with the pandemic limited the recruitment for this study resulting s small samples in some countries. This must be considered as a limitation. Further, our study cannot be considered as generalizable to the wider forensic population. About 30% of the potentially eligible patients refused to participate, and in line with ethical standards, we were not allowed to collect any data on them. Thus, we could not determine whether the refusers differed from those who were recruited into the study.

However, the use of multivariate analyses is a strength of this paper compared to some studies reporting only bivariate analyses [15, 29]. Nevertheless, due to the fact that we used a cross-sectional design we must not exclude reverse causality (e.g. association between unmet needs and symptom scores). But, an important strength of this study is the fact, that this is the first one applying the same methodology in forensic settings in different European countries.

## Conclusions

Our study shows that patients and staff frequently do not identify the same needs, and their assessments were influenced by different independent variables. It has been repeatedly shown that there are discrepancies in the amount and type of needs identified by patients and staff members in both non-forensic [38] and forensic settings [24, 34]. There are many reasons why the clinicians’ evaluations are important along with the subjective view of the patient. Forensic clinicians have extensive knowledge about which interventions might be appropriate for each specific patient in certain situations. However, patients sometimes refuse possibly helpful interventions for a wide variety of reasons. In such cases, sometimes better communication and information might lead to the acceptance of an intervention that was previously rejected. However, the needs recognised by staff must be better informed by patients’ wishes and experiences. Interventions that address the wishes and expectations of the affected person will have a better chance of being accepted and used, thus of being effective. We must not forget that when a health worker states the need for an intervention which the patient does not want to use, this need cannot be met. For this reason, it is essential to assess the subjective view of each affected person in addition to that of professionals [11].

It has been reported that failing to recognize unmet patient needs can lead to a poorer quality of life [39, 40]. O’Hara and colleagues [41] reported that forensic patients with depressive symptoms reported higher numbers of unmet needs. Krautgartner et al. [42] found that unmet needs of family caregivers were significantly associated with caregiver’s depression. Since this finding was based on a cross-sectional study, Krautgartner et al. [42] discussed if unmet needs cause depression or if depression hinder caregivers to seek help to meet their needs. Whether unmet needs contribute to poorer therapeutic outcomes and an increased risk of violent reoffending is still an open scientific question.

Considering all these aspects, the present study yielded important findings, but prospective cohort studies would be essential to complement our understanding of the interaction between needs and clinical and social aspects such as depression, quality of life, and repeat offence. Such prospective studies could eliminate reverse causality. Studies including samples from different countries should select countries on specific criteria such as numbers of forensic beds, numbers of general psychiatric beds or length of stay in forensic services.

## Supplementary Information

Below is the link to the electronic supplementary material.

Supplementary file1: **Table S1**. Forensic facilities recruiting for the present study. **Table S2**. Sample description split by country (SD = standard deviation). **Table S3**. Mean numbers of met, unmet and total needs by variables used for sample description (SD = standard deviation)

## Data Availability

The project will fully embrace the open access data policy of H2020 to make data FAIR (Findable, Accessible, Interoperable, and Re-usable), and all data gathered in the framework of the project are stored in a public repository (https://doi.org/10.5281/zenodo.4442372) accessible to all scientists willing to carry out additional analyses.
