# Supplementary material for: Needs of forensic psychiatric patients with schizophrenia in five European countries
Source: Soc Psychiatry Psychiatr Epidemiol. 2022 Jul 15;58(1):53–63. doi: 10.1007/s00127-022-02336-5 (PMC9284498; doi:10.1007/s00127-022-02336-5)
Supplement: Supplementary file 1 — Supplementary file1: Table S1. Forensic facilities recruiting for the present study. Table S2. Sample description split by country (SD = standard deviation). Table S3. Mean numbers of met, unmet and total needs by variables used for sample description (SD = standard deviation) [file 127_2022_2336_MOESM1_ESM.docx]

**Table S1: Forensic facilities recruiting for the present study**

| **Country** | **Name od facility and location** | **Number of forensic beds** |
| --- | --- | --- |
| Austria | Justizanstalt Goellersdorf, Lower Austria, Goellersdorf | 145 |
|  | Klinik für Psychiatrie mit forensischem Schwerpunkt, Upper Austria, Linz | 53 |
| Germany | Klinik für Forensische Psychiatrie und Psychotherapie, Zentrum für Psychiatrie Nordbaden, Wiesloch | 258 |
|  | Klinik für Forensische Psychiatrie des Pfalzklinikums, Klingenmünster | 185 |
|  | Klinik für Forensische Psychiatrie und Psychotherapie, Weinsberg | 124 |
| Italy | REMS, Centro Polifunzionale ex Ospedale Stellini, ULSS 9 ‘Scaligera’, Nogara | 130 |
|  | Sistema Polimodulare REMS, Castiglione delle Stiviere | 20 |
|  | REMS Minerva, ASL Roma 5, Rome | 20 |
|  | REMS-D, Azienda USL Toscana Nord-Ovest, Volterra | 20 |
| Poland | Institute of Psychiatry and Neurology, Department of Forensic Psychiatry, Warsaw | 60 |
|  | SP ZOZ Psychiatryc Hospital, Department of Forensic Psychiatry, Toszek, | 40 |
|  | Psychiatric Hospital, Forensic Department, Starogard Gdański | 40 |
| England | South London and Maudsley NHS Foundation Trust, London | 130 |
|  | South West London and St. Georges Mental Health NHS Trust, London | 75 |
|  | St. Andrew’s Healthcare | 240 |

**Table S2:** Sample description split by country (SD = standard deviation)

|  | **Austria** | **Germany** | **Italy** | **Poland** | **England** |
| --- | --- | --- | --- | --- | --- |
|  |  |  |  |  |  |
| Sex |  |  |  |  |  |
| Male | 92,0% | 83,3% | 94,9% | 85,7% | 85,0% |
| Female | 8,0% | 16,7% | 5,1% | 14,3% | 15,0% |
| Age (years) |  |  |  |  |  |
| Mean (SD) | 39.66 (11.32) | 38.06 (11.14) | 39.54 (11.73) | 40.14 (12.02) | 38.10 (9.57) |
| Highest occupational status |  |  |  |  |  |
| Unskilled | 60,0% | 61,1% | 87,2% | 51,8% | 77,5% |
| Skilled / professional | 40,0% | 38,9% | 12,8% | 48,2% | 22,5% |
| Type of Schizophrenia Spectrum Disorder |  |  |  |  |  |
| Schizophrenia | 82,0% | 91,7% | 56,4% | 89,3% | 70,0% |
| Other | 18,0% | 8,3% | 43,6% | 10,7% | 30,0% |
| PANSS positive score |  |  |  |  |  |
| Mean (SD) | 15,61 (7,71) | 11,86 (4,52) | 13,21 (5,49) | 16,29 (7,33) | 15,90 (7,36) |
| PANSS negative score |  |  |  |  |  |
| Mean (SD) | 18,53 (9,24) | 15,17 (6,03) | 18,31 (7,50) | 22,05 (7,62) | 18,85 (5,88) |
| PANSS general score |  |  |  |  |  |
| Mean (SD) | 34.91 (11.80) | 27.17 (6.96) | 33.59 (10.18) | 38.96 (12.88) | 31.92 (8.68) |
| Comorbidity with personality disorder |  |  |  |  |  |
| No | 66,7% | 76,5% | 61,5% | 82,1% | 63,2% |
| Yes | 33,3% | 23,5% | 38,5% | 17,9% | 36,8% |
| Lifetime substance or alcohol use |  |  |  |  |  |
| No | 24,0% | 19,4% | 28,2% | 30,4% | 7,5% |
| Yes | 76,0% | 80,6% | 71,8% | 69,6% | 92,5% |
| Lifetime ever attempted suicide(s) or self-harm |  |  |  |  |  |
| No | 48,0% | 52,8% | 74,4% | 55,4% | 41,0% |
| Yes | 52,0% | 47,2% | 25,6% | 44,6% | 59,0% |
| Duration of illness (years) |  |  |  |  |  |
| Mean (SD) | 14.22 (11.42) | 12.38 (7.31) | 10.92 (9.28) | 12.44 (9.67) | 16.01 (8.49) |
| Type of crime (index offence) |  |  |  |  |  |
| (Attempted) homicide | 42,0% | 44,4% | 79,5% | 48,2% | 22,5% |
| Other | 58,0% | 55,6% | 20,5% | 51,8% | 77,5% |
| History of any other violent behaviour lifetime (in addition to index offence) |  |  |  |  |  |
| No | 22,0% | 38,2% | 66,7% | 32,7% | 5,1% |
| Yes | 78,0% | 61,8% | 33,3% | 67,3% | 94,9% |
| Age at first contact with mental health services (years) |  |  |  |  |  |
| Mean (SD) | 23.36 (9.56) | 25.71 (9.11) | 28.72 (9.29) | 26.64 (9.62) | 20.7 (4.85) |
| Time since first admission to a forensic unit (years) |  |  |  |  |  |
| Mean (SD) | 7.00 (7.64) | 6.33 (6.55) | 3.97 (4.50) | 4.55 (5.58) | 9.65 (8.47) |
| Number of lifetime admissions to forensic units |  |  |  |  |  |
| Mean (SD) | 1.67 (1.05) | 1.08 (0.37) | 1.05 (0.22) | 1.18 (0.43) | 2.55 (2.33) |

**Table S3:** Mean numbers of met, unmet and total needs by variables used for sample description (SD = standard deviation)

|  |  | **Patient** | | | | | | **Staff** | | | | | | |
| --- | --- | --- | --- | --- | --- | --- | --- | --- | --- | --- | --- | --- | --- | --- |
|  |  | **Met needs** | | **Unmet needs** | | **Total needs** | | **Met needs** | | **Unmet needs** | | **Total needs** | | |
|  |  | **Mean** | **SD** | **Mean** | **SD** | **Mean** | **SD** | **Mean** | **SD** | **Mean** | **SD** | **Mean** | **SD** |  |
|  |  |  |  |  |  |  |  |  |  |  |  |  |  |  |
| Sex | Male | 3.72 | 2.06 | 2.64 | 2.73 | 6.35 | 3.67 | 4.72 | 2.82 | 2.02 | 2.16 | 6.74 | 3.66 |  |
|  | female | 3.88 | 2.42 | 1.54 | 2.23 | 5.42 | 3.87 | 6.46 | 3.18 | 1.85 | 1.91 | 8.31 | 3.61 |  |
| Age (Years) | 18-34 | 3.59 | 1.81 | 2.33 | 2.15 | 5.92 | 3.20 | 4.91 | 2.85 | 1.76 | 1.85 | 6.67 | 3.54 |  |
|  | 35-49 | 3.91 | 2.12 | 2.87 | 3.19 | 6.78 | 3.97 | 4.74 | 2.97 | 2.23 | 2.39 | 6.98 | 3.85 |  |
|  | 50-65 | 3.71 | 2.51 | 2.17 | 2.58 | 5.88 | 3.99 | 5.27 | 2.95 | 2.02 | 2.11 | 7.29 | 3.67 |  |
| Highest occupational status | Unskilled | 3.49 | 2.37 | 2.09 | 2.64 | 5.59 | 3.79 | 4.47 | 3.03 | 1.81 | 2.20 | 6.28 | 3.85 |  |
|  | Skilled / professional | 3.86 | 1.94 | 2.72 | 2.70 | 6.58 | 3.61 | 5.16 | 2.83 | 2.10 | 2.09 | 7.25 | 3.56 |  |
| Type of Schizophrenia Spectrum Disorder | Schizophrenia | 3.70 | 2.09 | 2.28 | 2.49 | 5.97 | 3.51 | 4.84 | 2.93 | 2.03 | 2.03 | 6.88 | 3.63 |  |
|  | Other | 3.89 | 2.13 | 3.36 | 3.21 | 7.26 | 4.18 | 5.21 | 2.87 | 1.87 | 2.47 | 7.09 | 3.92 |  |
| PANSS positive score | < 12 | 3.66 | 1.98 | 2.13 | 1.99 | 5.80 | 3.01 | 4.46 | 2.93 | 1.30 | 1.58 | 5.76 | 3.33 |  |
|  | > 13 | 3.88 | 2.16 | 2.89 | 3.17 | 6.77 | 4.14 | 5.35 | 2.86 | 2.60 | 2.36 | 7.95 | 3.69 |  |
| PANSS negative score | < 18 | 3.83 | 2.10 | 2.41 | 2.53 | 6.25 | 3.41 | 4.55 | 2.86 | 4.55 | 2.86 | 6.06 | 3.52 |  |
|  | > 19 | 3.73 | 2.04 | 2.64 | 2.87 | 6.38 | 3.94 | 5.34 | 2.93 | 5.34 | 2.93 | 7.84 | 3.65 |  |
| PANSS general score | < 31 | 3.67 | 1.97 | 2.17 | 2.33 | 5.84 | 3.23 | 4.43 | 2.85 | 1.39 | 1.69 | 5.82 | 3.53 |  |
|  | > 32 | 3.94 | 2.19 | 2.97 | 3.03 | 6.91 | 4.07 | 5.53 | 2.79 | 2.61 | 2.32 | 8.15 | 3.45 |  |
| Comorbidity with personality disorder | No | 3.72 | 2.08 | 2.54 | 2.80 | 6.26 | 3.77 | 4.87 | 2.76 | 2.12 | 2.20 | 6.99 | 3.66 |  |
|  | Yes | 3.79 | 2.22 | 2.35 | 2.30 | 6.14 | 3.48 | 5.25 | 3.12 | 1.76 | 1.97 | 7.02 | 3.47 |  |
| Lifetime substance or alcohol use | No | 3.90 | 2.46 | 1.90 | 2.23 | 5.80 | 3.83 | 5.02 | 3.13 | 1.84 | 2.01 | 6.86 | 3.80 |  |
|  | Yes | 3.69 | 1.99 | 2.70 | 2.79 | 6.39 | 3.66 | 4.87 | 2.85 | 2.05 | 2.17 | 6.92 | 3.66 |  |
| Lifetime ever attempted suicide(s) or self-harm | No | 3.62 | 2.03 | 2.08 | 2.16 | 5.71 | 3.34 | 5.01 | 2.86 | 1.76 | 1.93 | 6.77 | 3.62 |  |
|  | Yes | 3.87 | 2.18 | 2.95 | 3.12 | 6.82 | 3.98 | 4.83 | 2.99 | 2.28 | 2.33 | 7.11 | 3.78 |  |
| Duration of illness (Years) | 0-10 | 3.64 | 1.91 | 2.20 | 2.05 | 5.84 | 3.12 | 5.03 | 3.09 | 1.78 | 1.82 | 6.81 | 3.65 |  |
|  | 11-20 | 3.84 | 2.08 | 2.71 | 3.13 | 6.56 | 4.12 | 4.84 | 2.79 | 2.00 | 2.31 | 6.84 | 3.64 |  |
|  | 21-41 | 3.93 | 2.54 | 2.90 | 3.18 | 6.83 | 4.16 | 5.05 | 2.56 | 2.64 | 2.41 | 7.69 | 3.52 |  |
| Type of crime (index violence) | (Attempted) homicide | 3.84 | 2.21 | 2.08 | 2.31 | 5.91 | 3.40 | 5.23 | 3.05 | 1.61 | 1.89 | 6.84 | 3.62 |  |
|  | Other | 3.65 | 2.00 | 2.89 | 2.94 | 6.54 | 3.93 | 4.65 | 2.77 | 2.35 | 2.28 | 7.00 | 3.76 |  |
| History of any other violent behaviour lifetime (in addition to index offence) | No | 3.36 | 2.04 | 1.80 | 1.98 | 5.16 | 3.40 | 4.76 | 2.97 | 1.24 | 1.65 | 6.00 | 3.52 |  |
|  | Yes | 3.92 | 2.11 | 2.83 | 2.91 | 6.75 | 3.73 | 5.09 | 2.87 | 2.39 | 2.25 | 7.48 | 3.63 |  |
| Age at first contact with mental health services (Years) | 0-24 | 3.77 | 2.06 | 2.85 | 2.83 | 6.62 | 3.81 | 4.92 | 2.65 | 2.20 | 2.11 | 7.12 | 3.41 |  |
|  | 25-60 | 3.78 | 2.16 | 2.12 | 2.53 | 5.90 | 3.58 | 4.99 | 3.09 | 1.76 | 2.10 | 6.74 | 3.71 |  |
| Time since first admission to a forensic unit (Years) | 0-10 | 3.75 | 2.03 | 2.32 | 2.45 | 6.07 | 3.55 | 4.99 | 2.96 | 1.88 | 1.99 | 6.86 | 3.73 |  |
|  | > 11 | 3.81 | 2.37 | 3.36 | 3.35 | 7.17 | 4.10 | 4.57 | 2.59 | 2.57 | 2.53 | 7.15 | 3.53 |  |
| Number of lifetime admissions to forensic units | 1 | 3.63 | 2.09 | 2.20 | 2.36 | 5.83 | 3.45 | 4.79 | 2.90 | 1.95 | 2.05 | 6.74 | 3.67 |  |
|  | > 2 | 4.06 | 2.09 | 3.46 | 3.36 | 7.52 | 4.14 | 5.33 | 2.92 | 2.15 | 2.39 | 7.48 | 3.67 |  |
